# Pulse Driven Injection in an Additive-Manufactured Microchannel for Rapid Mixing of Stratified Concurrent Flow and On-Demand Droplet Generation

**DOI:** 10.3390/mi17050540

**Published:** 2026-04-28

**Authors:** Faisal bin Nasser Sarbaland, Masashi Kobayashi, Daiki Tanaka, Risa Fujita, Nobuyuki Tanaka, Masahiro Furuya

**Affiliations:** Faculty of Advanced Science and Engineering, Waseda University, Tokyo 162-0041, Japan; faisaru@fuji.waseda.jp (F.b.N.S.); maajii@fuji.waseda.jp (M.K.); d.tanaka@ruri.waseda.jp (D.T.); r.fujita@aoni.waseda.jp (R.F.); nobuyuki_tanaka@aoni.waseda.jp (N.T.)

**Keywords:** microfluidics, additive manufacturing, pulse-driven injection, pressure actuation, micromixing, droplet-on-demand, water-in-oil droplets

## Abstract

Laminar co-flow in microchannels typically results in stratified streams with diffusion-limited mixing. This work presents an additively manufactured microfluidic platform that integrates a pulse tank and a transverse injection nozzle into an otherwise straight channel, enabling pulse-driven mixing and droplet generation using air-pressure actuation alone. In Device A, transverse pulsed injection disrupted the stratified interface and significantly enhanced mixing compared with the no-pulse case, as confirmed by an entropy-based mixing index. In Device B, pulsed injection into a continuous oil phase enabled stable droplet-on-demand generation with pressure-tunable droplet diameter in a straight circular channel. The devices operated in a laminar regime, with representative Reynolds, Péclet, and capillary numbers confirming diffusion-limited baseline mixing and stable dripping-type droplet formation. The results demonstrate that pulse-driven injections in a simple, additively manufactured geometry provide an effective, low-complexity approach to mixing enhancement and droplet generation without external fields or complex channel designs.

## 1. Introduction

Laminar flow in microchannels is characterized by smooth, orderly fluid motion, which significantly limits mixing efficiency due to the dominance of viscous forces and the restricted interaction between fluid layers [1]. As a result, mixing primarily occurs through diffusion, leading to slow mixing rates in microfluidic systems [2]. This slow mixing can adversely affect reaction rates and the overall performance of microreactors and lab-on-a-chip applications, where efficient mixing is crucial for optimizing biological and chemical processes [3]. Rapid mixing is essential in these applications because it enhances reactant interactions, thereby improving reaction outcomes and efficiency, particularly in processes such as genetic analysis and disease diagnosis [4]. Additionally, controllable droplet generation allows for precise manipulation of reaction conditions and the formation of discrete reaction environments, which is vital for achieving optimal results in microfluidic systems [5]. By facilitating rapid mixing and enabling the generation of uniform droplets, these techniques help overcome the limitations imposed by laminar, stratified concurrent flows, ultimately leading to enhanced mixing efficiency and improved system performance [6]. Therefore, integrating effective mixing strategies and droplet generation methods is critical for advancing the capabilities of microreactors and lab-on-a-chip technologies [7].

Microfluidic mixing and droplet generation techniques are essential for various applications, with passive and active mixers being prominent approaches. Passive mixers, such as serpentine and herringbone designs, rely solely on pumping energy for mixing, which can limit their efficiency and controllability because they lack external energy sources. These mixers often feature geometries that enhance mixing via chaotic advection and flow impingement, but they may struggle to achieve optimal mixing over short distances [1]. Active mixers, on the other hand, utilize external energy sources, such as electrokinetic or ultrasound forces, to enhance mixing efficiency and control. However, this increased performance comes at the cost of greater fabrication complexity, as active mixers require more intricate designs and components [8]. Droplet generation techniques, including T-junctions and flow-focusing droplet generators, also present unique challenges. T-junctions create droplets by splitting a continuous phase, but controlling droplet size and formation can be difficult. Flow-focusing generators, which concentrate a continuous phase around a dispersed phase, face similar precision challenges due to flow-rate variations. Both methods can complicate fabrication and control, particularly when precise droplet formation is necessary [9]. In summary, passive and active mixers, as well as droplet generation techniques, each have limitations related to fabrication complexity and controllability that must be addressed for practical application in microfluidic systems [1,10].

Current literature highlights a significant gap in achieving fast mixing and tunable droplet generation within a simple, straight microchannel, particularly when utilizing additively manufactured devices [11,12,13,14,15,16]. While fast mixing is essential for enhancing reaction rates and ensuring uniformity in droplet generation, existing methods often rely on complex geometries or external fields, which may not be feasible in all applications [17]. Furthermore, the ability to dynamically adjust droplet size and volume during formation is crucial for specific applications, yet this capability is often limited by the design constraints of traditional microfluidic systems [5]. The integration of pressure-based pulse control offers a promising approach to generate droplets on demand, allowing for precise control over droplet characteristics without the need for external fields [18]. However, the challenge remains to implement this technique effectively in a straightforward microchannel design that facilitates both rapid mixing and tunable droplet generation. The flow-focusing geometry, while effective in enhancing mixing and droplet formation, may not fully exploit the potential of pressure-based methods in a simple configuration [19]. Addressing these gaps could lead to significant advances in microfluidics, particularly in applications that require precise droplet manipulation.

Pulse-driven perturbation and pressure-actuated droplet generation have been explored in prior microfluidic systems, and conventional T-junction and flow-focusing geometries remain widely used baselines for droplet production. Likewise, 3D-printed droplet generators have been demonstrated in co-flow and cross-flow formats and can achieve low polydispersity under appropriate flow conditions [11,14,16], while active/on-demand droplet generation concepts have been broadly reviewed [5]. The contribution of the present study is therefore not pulsing per se, but a straight-channel, monolithic additively manufactured implementation that integrates a pulse tank and transverse nozzle to realize two functions with the same pneumatic actuation architecture: pulse-triggered mixing enhancement of stratified co-flow (Device A) and droplet-on-demand generation using a single continuous-phase flow and pulsed dispersed-phase injection (Device B). To support comparison and make the “simplicity/effectiveness” claims quantitative, Table 1 benchmarks the present platform against representative conventional, 3D-printed, and pulse/active droplet systems using several practical performance and implementation characteristics.

In this work, we present an additively manufactured microfluidic platform that integrates a pulse tank and a transverse injection nozzle into an otherwise straight microchannel. Using the same pneumatic actuation concept, the platform operates in two configurations: Device A for pulse-triggered mixing enhancement in stratified co-flow, and Device B for droplet-on-demand generation in a continuous oil phase. This study aims to evaluate whether this simple straight-channel architecture can provide effective mixing enhancement and controllable droplet formation without external fields or complex channel geometries.

## 2. Method

### 2.1. Devices

The present platform uses a pulse-driven injection concept integrated into an otherwise straight additively manufactured microchannel. A side pulse tank is connected to the main channel through a small orifice, and short air-pressure pulses from this tank inject fluid transversely into the main flow. The concept was implemented in two configurations. In Device A, pulsed injection occurs between two aqueous streams to perturb the stratified interface and enhance mixing over a much shorter downstream distance than in diffusion-limited co-flow. In Device B, the same pulse-injection concept is used to inject an aqueous phase into a continuous oil phase within a circular channel, thereby generating water-in-oil droplets on demand. In both configurations, the geometry remains simple and fully additively manufactured, while the response is controlled primarily by the applied pulse pressure and pulse duration.

**Figure 1:**
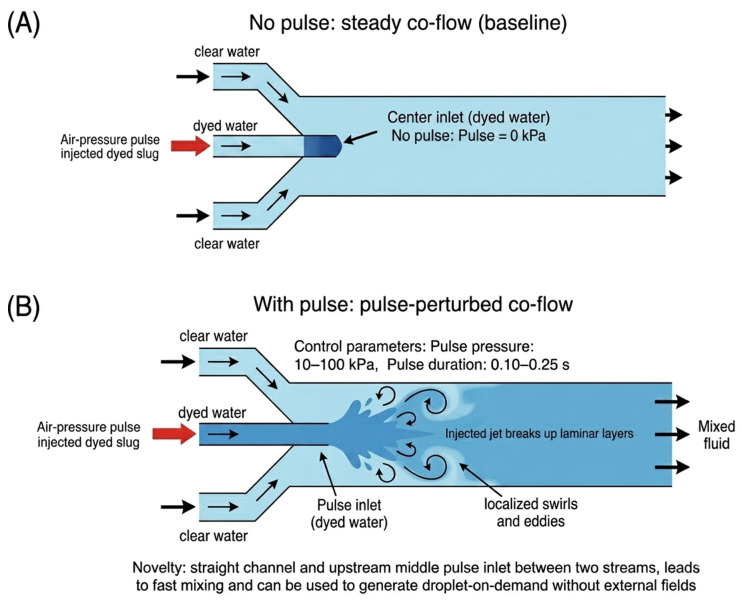
Flow-state definitions for pulse-driven injection in Device A: (**A**) no-pulse stratified concurrent flow; (**B**) pulse-perturbed flow produced by a transverse air-pressure injection.

Figure 1 defines the two flow states used in Device A: (A) a no-pulse baseline stratified concurrent flow formed by blue-dyed and transparent water streams entering from the left and right inlets, and (B) a pulse-perturbed flow produced by applying a short air-pressure pulse to the pulse tank to inject a dyed slug through the transverse nozzle. These two states are later compared in the Results section using visual mixing length and an entropy-based mixing index (MI). The same pulse-driven injection principle is also applied to droplet-on-demand generation in Device B.

All microfluidic devices in this study were fabricated by additive manufacturing using a Keyence AGILISTA-3200W 3D printer (Keyence Corp., Tokyo, Japan) with the transparent AR-M2 photopolymer resin (Keyence Corp., Tokyo, Japan).

To evaluate the dimensional fidelity of the printed devices, accessible channel openings (inlets and outlets) were measured using optical microscopy on three independently printed devices (*n* = 3). The measured diameters were compared with the CAD values. The printed channels showed a consistent undersizing relative to the CAD design with an average deviation of approximately 175 µm (range 150–200 µm). Table 2 summarizes the measured channel diameters compared with the CAD design values. The measurements were performed on three independently printed devices, providing an estimate of batch-to-batch dimensional variation of the printing process.

The device geometries, including the pulse tank, injection nozzle seat, and straight main channels, were exported as STL files and printed as single, monolithic parts. Printing was carried out in the printer’s high-resolution mode, corresponding to a nominal layer thickness of approximately 15 µm for AR-M2, and the devices were oriented so that the main channels were roughly parallel to the build platform to reduce stair-stepping on the channel walls and to keep support material away from the smallest internal features. After printing, the parts were rinsed in water, dried with compressed air, and inspected under a microscope to verify that all channels and the nozzle seat were open before the experiments. Because the printed devices were not sectioned destructively, the true internal cross-sectional geometry and internal wall roughness were not measured directly; fabrication characterization in this study was therefore limited to measurements of accessible openings and optical inspection of the as-built channels.

Previous studies using similar droplet-based photopolymer printing processes have shown that surface quality depends on printer type, build orientation, and printing parameters [22,23]. These literature reports are provided only as general background and should not be interpreted as direct roughness measurements of the present devices.

**Figure 2:**
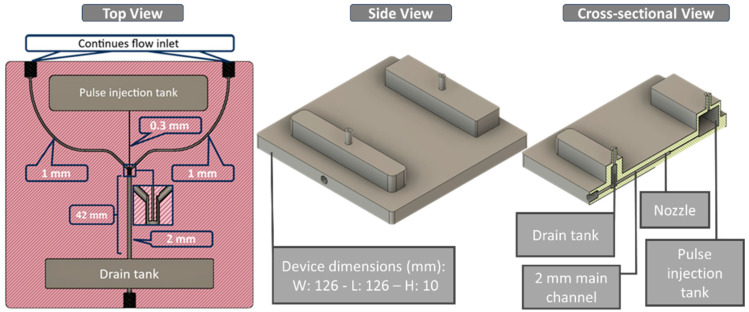
Device A for mixing. Top and side views showing the pulse tank, transverse injection nozzle, straight main channel, and side inlets.

For Device A (Figure 2), the mixing geometry consists of a single-piece printed body with an upstream pulse tank, two side inlets, a straight main channel, and a downstream drain tank. The pulse tank holds the dyed aqueous phase and feeds a resin-printed injection nozzle with an inner diameter of 0.3 mm. This nozzle connects to a straight main channel with an inner diameter of 2.0 mm, forming a locally expanded region where the injected jet meets the main flow. Transparent water enters the device through two symmetric side inlets on the left and right. Each side inlet has an inner diameter of 1.0 mm and connects to the main channel just upstream of and aligned with the nozzle junction, ensuring that the two streams meet at the injection point. Downstream of this junction, the main channel continues straight to the outlet and drain tank. Under the baseline operating condition of 500 µL/min without pulsing, the interface between the dyed and transparent streams persists for approximately 42 mm along the channel. When a single 10 kPa, 0.250 s pulse is applied to the pulse tank, the injected jet from the 0.3 mm nozzle disrupts this interface. It produces rapid mixing, with the dyed and transparent streams appearing visually uniform within about 1.5 mm downstream of the junction. The overall printed body measures approximately 126 mm in length, 126 mm in width, and 10 mm in height, with the pulse tank located on the nozzle side and a drain tank at the outlet end.

**Figure 3:**
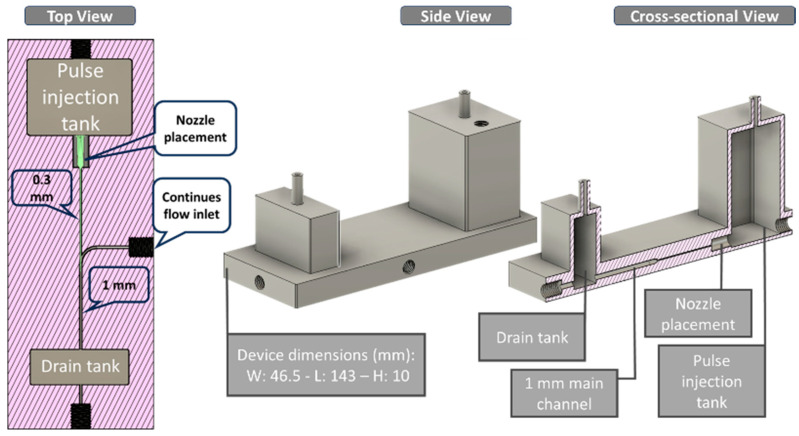
Device B for droplet generation. Top and side views of the straight circular main channel with a transverse stainless-steel injection nozzle.

Device B (Figure 3) uses the same AR-M2 printed body thickness (≈10 mm) but a different channel layout optimized for water-in-oil droplet formation. The printed main channel is a straight, circular cross-section channel (1.0 mm inner diameter) running along the long axis of the device. A stainless-steel capillary nozzle with an inner diameter of 150 µm and an outer diameter of 200 µm was press-fitted into a side injection port on the pulse-tank side and terminated near the channel centerline without contacting the opposite wall of the main channel. In this final position, the nozzle axis is approximately perpendicular to the central channel axis, and its outlet faces directly into the 1.0 mm-diameter oil stream. Accordingly, a continuous annular clearance remains around the nozzle tip, allowing the oil to flow past the tip without obstruction. The insertion depth is therefore set by the wall thickness at the injection point and was kept constant in all experiments. The overall printed body measures approximately 143 mm long, 46.5 mm wide, and 10 mm high, with the pulse tank on the nozzle side and the drain tank at the outlet end. Water from the pulse tank passes through the stainless-steel nozzle and is injected into the continuous canola oil flowing along the main channel, thereby forming droplets at the nozzle orifice within the circular channel.

The stainless-steel nozzle in Device B was mounted from the pulse-tank side into a cylindrical port printed in the device body. Before insertion, a thin layer of transparent silicone sealant (Shin-Etsu KE45T-100, Universal Sealant, Shin-Etsu Chemical Co., Ltd., Tokyo, Japan) was applied along the outer surface of the nozzle body. The port diameter was designed with a small clearance relative to the nozzle outer diameter so that the nozzle could be inserted smoothly with the sealant and pushed in until its tip reached the inner wall of the 1.0 mm main channel, with the nozzle axis approximately perpendicular to the channel. After positioning, any excess sealant at the outer surface was gently removed, leaving a continuous fillet around the junction between the steel tube and the printed resin. The sealant was then allowed to cure fully at room temperature before use. Before each experiment, the assembled device was checked for leakage by filling the main channel with liquid, applying air pressure to the pulse tank up to (or slightly above) the maximum experimental value, and visually inspecting the nozzle region and external joints under a stereomicroscope to confirm that no bubbles or seepage were present. The channel sizes were selected to ensure a clear laminar baseline co-flow while keeping all internal features reliably manufacturable in transparent AR-M2 resin. For Device A, the main channel diameter is 2.0 mm. The total flow rate is 500 µL/min, giving a mean velocity on the order of 10^−3^ m/s and a Reynolds number, Re = ρUD/μ, on the order of 1–10 for water at room temperature, i.e., a stable laminar regime where mixing is diffusion-limited. This motivates transverse pulsed injection: a short jet-like perturbation can create strong cross-stream transport and rapid interfacial deformation without requiring long chaotic-mixer geometries or external fields. The 0.3 mm injection orifice was chosen as a practical minimum size that remains open after printing/cleaning, while still much smaller than the main channel, to generate a localized transverse disturbance. For Device B, the main channel diameter was reduced to 1.0 mm to increase confinement and stabilize dripping. A 150 µm-ID stainless-steel nozzle was used to provide a well-defined orifice that is difficult to reproduce by resin printing alone. Overall, the geometry balances baseline laminar operation, strong localized perturbation during pulses, and robust fabrication/assembly; more detailed CFD-based optimization is left for future work.

### 2.2. Fluids

For all experiments, we used purified water as the aqueous phase and commercial canola oil as the oil phase. In the mixing experiments with Device A, both streams were water. For the no-pulse reference, blue-dyed water was supplied through the left inlet and transparent water through the right inlet, producing a visible stratified concurrent flow. In the single-pulse condition, both side inlets supplied transparent water, while the pulse tank contained blue-dyed water injected through the transverse nozzle only during the pulse. Unless otherwise noted, we focus on a reference condition in which the two side streams were driven at a total flow rate of 500 µL/min, and a single 10 kPa, 0.250 s pressure pulse was applied to the pulse tank. In the droplet-generation experiments with Device B, the pulse tank was filled with water, and the continuous phase was canola oil driven by the syringe pump through the 1.0 mm main channel. The oil flow rate was set to 200 µL/min, while the water injection pressure varied from 30 to 100 kPa, with a fixed pulse duration of 0.10 s and a vacuum holdback of 1.1 kPa to control the volume injected per pulse and hence the droplet size. All experiments were carried out at room temperature (approximately 22 °C) without additional temperature control. No surfactants were added to either phase. Because the interfacial properties of purified water and commercial canola oil can vary with source, lot, and temperature, all experiments were performed at room temperature and under consistent handling and imaging conditions to ensure comparability across runs.

The relevant physical properties of the working fluids at room temperature (≈22 °C) are summarized in Table 3. The densities of the actual purified water and commercial canola oil used in the experiments were measured gravimetrically across three independent batches for each liquid, and the reported values are mean ± standard deviation. For purified water, the dynamic viscosity was estimated from a standard temperature–viscosity correlation using the experimental temperature. For canola oil, a representative literature value at approximately 22 °C was used. The water–oil interfacial tension was likewise taken as a representative literature value based on the reported range for commercial vegetable oils with water [24,25,26,27]. Accordingly, the values in Table 3 include both directly measured and literature- and correlation-based properties, and the resulting dimensionless numbers should be interpreted as approximate indicators of operating regimes.

### 2.3. Setup

**Figure 4:**
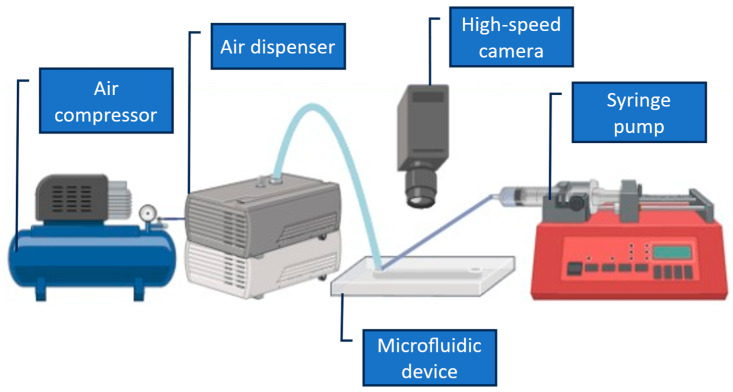
Experimental setup for pulse-driven mixing and droplet generation.

The experimental setup is shown in Figure 4. Compressed air from an oil-less compressor (EARTH MAN ACP-13SLA, 13 L, Takagi Co., Ltd., Sanjo, Japan) was supplied to a pneumatic dispenser (Musashi Engineering ML-5000XII, Musashi Engineering, Inc., Tokyo, Japan), which set the injection pressure, vacuum holdback, and dispense time for the pulse tank. In the experiments reported here, the injection pressure was typically adjusted between 10 and 100 kPa, with a vacuum holdback of 1.1 kPa in the droplet experiments. Single pulses were generated either by pressing the “SHOT” button on the dispenser or by triggering the dispenser electronically using a function generator (AS ONE AWG1005, AS ONE Corporation, Osaka, Japan), which defined the pulse duration and frequency. The pulse duration was set to 0.250 s for the mixing experiments (Device A) and 0.10 s for the droplet-generation experiments (Device B). Liquid flow rates were controlled by a syringe pump (KD Scientific syringe pumps, Legato 180, KD Scientific, Inc., Holliston, MA, USA) fitted with 50 mL disposable syringes (Terumo), Terumo Corporation, Tokyo, Japan via PTFE tubing (inner diameter 1.0 mm) in Device A. The pump delivered a total of 500 µL/min of purified water to the two side inlets. At the same time, Device B supplied canola oil at 200 µL/min through the 1.0 mm main channel. The devices were mounted on an upright microscope (SVBONY) and observed with a SWIFTCAM SC2, SVBONY Optics Technology Co., Ltd., Shenzhen, Guangdong, China 503 USB camera, which was used to record images, Swift Optical Instruments, Inc., San Jose, CA, USA and short videos at 15 frames/s of the mixing and droplet-generation regions for subsequent analysis. The pulse conditions used in this study were selected empirically to provide stable, repeatable operation while remaining within safe limits for the printed resin body and sealing interfaces. For Device A, a pulse duration of 0.250 s at relatively low pressure (typically 10–40 kPa) was chosen because it produced a clearly observable, single-shot transverse perturbation without causing leakage, bubble intrusion, or mechanical stress at the pulse-tank junction. For Device B, a shorter pulse duration (0.10 s) was selected to promote single-droplet pinch-off per pulse and to avoid multi-droplet ejection during a single actuation event. The upper pressure range was limited to prevent over-pressurization of the pulse tank and to maintain stable dripping at the nozzle without transitioning to a jetting-like behavior. Overall, the reported settings represent a practical “stable window” for the present prototypes; a full optimization of pressure and pulse width is left for future work.

### 2.4. Analysis

The mixing length was defined as the downstream distance from the nozzle exit (x = 0) to the point at which the dyed/transparent interface was no longer visually distinguishable in microscopy images. This visual measure was used as an intuitive indicator of mixing behavior, while quantitative mixing performance was evaluated using an entropy-based mixing index derived from image analysis. Distances were measured on recorded microscopy images using a pixel-to-length calibration based on the known 2.0 mm inner diameter of the main channel. In Device B, droplet size was characterized by measuring the equivalent circular diameter of individual water-in-oil droplets after pinch-off, a short distance downstream of the nozzle. For each injection pressure, images were selected from the recorded videos after a stable droplet formation pattern had been established. The apparent diameters of individual droplets were measured using the known 1.0 mm inner diameter of the main channel as a geometric reference. For each pressure condition, ten droplets were measured from each device, and the resulting measurements were combined across independently printed devices (*n* = 3). The mean droplet diameter and standard deviation were calculated from the resulting dataset.

An uncertainty analysis was performed to evaluate the repeatability of the measurements. For the mixing experiments, the entropy-based mixing index was first computed for each detected pulse and then averaged across devices. The reported values correspond to the mean and standard deviation across independently printed devices (*n* = 3). For droplet-generation experiments, droplet diameters were measured from microscopy images using the known channel diameter as a geometric calibration reference. The image-based measurements were performed using calibrated microscopy images, with the pixel-to-length conversion determined from the known channel diameter. For each pressure condition, ten droplets were measured per device, and the dataset was combined across three independently printed devices (*n* = 30 droplets). The error bars shown in the figures represent the standard deviation of the measurements, reflecting pulse-to-pulse variation, device-to-device variation, and image measurement uncertainty.

To clarify the flow regime of the present system, representative dimensionless numbers were estimated using the measured geometry, flow conditions, and the fluid properties summarized in Table 3. The Reynolds number was calculated as Re = ρUD/μ, where ρ is the fluid density, U is the mean velocity in the main channel, D is the channel diameter, and μ is the dynamic viscosity [28]. The Péclet number was calculated as Pe = UD/Dm, where Dm is the molecular diffusion coefficient [29]. For droplet-generation experiments, the capillary number was estimated as Ca = μcUc/γ, where μc is the viscosity of the continuous phase, Uc is the continuous-phase velocity, and γ is the interfacial tension between the two fluids [30]. The viscosity ratio was calculated as λ = μd/μc, where μd and μc are the viscosities of the dispersed and continuous phases, respectively [31]. Because the properties in Table 3 include both directly measured and literature- and correlation-based values, the resulting dimensionless numbers should be interpreted as approximate indicators of operating regimes.

## 3. Results

### 3.1. Device Overview

The printed geometries of both Device A and Device B reproduced the CAD designs sufficiently for all experiments. The main straight channels, side inlets, pulse tank, and nozzle seats were all clearly formed in the AR-M2 resin, and no apparent warping or collapse of the 2.0 mm (Device A) and 1.0 mm (Device B) channels was observed under the microscope. The small injection orifices from the pulse tank into the main channels were open and well aligned with the channel centerline, allowing the dyed water jet in Device A and the stainless-steel nozzle in Device B to be positioned as designed. After inserting and sealing the metal nozzle in Device B, the assembled devices were pressurized to the maximum operating pressures used in this study (10–100 kPa at the pulse tank). At the same time, the main channel was filled with liquid. During these tests and throughout the mixing and droplet-generation experiments, we observed no visible leakage at the nozzle junction, the printed interfaces, or the external tubing connections, and no mechanical damage or cracking of the AR-M2 body.

**Figure 5:**
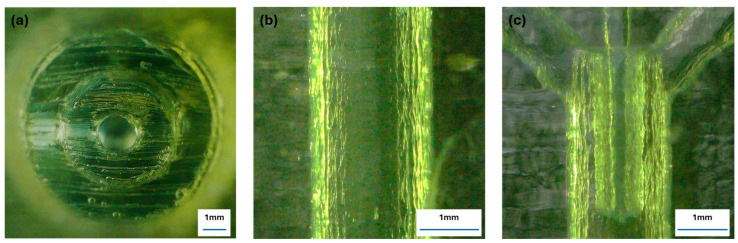
Optical images of the additively manufactured device showing the as-built channel geometry. (**a**) Outlet view of the main channel showing the approximately circular channel opening. (**b**) Longitudinal view of the printed channel wall showing the visible printed surface texture. This panel is qualitative only and is not intended as a quantitative roughness measurement. (**c**) View of the transverse injection region showing the nozzle aligned with the main channel. These images confirm channel openness and illustrate the printed geometry used in the experiments.

Representative optical images of the printed devices are shown in Figure 5. The images confirm that the main channel remained open after fabrication and post-processing. The outlet view shows an approximately circular channel opening, while the longitudinal view shows the visible printed surface texture of the channel wall. These images are qualitative only and are not intended to provide a quantitative measure of roughness. The junction image also indicates the alignment of the transverse nozzle with the main channel used for pulse-driven injection experiments. The outlet view also indicates limited ovality of the printed channel geometry.

The estimated dimensionless numbers are summarized in Table 4. For Device A, the Reynolds number (Re ≈ 5.5) indicates laminar flow conditions in the millimeter-scale channel. In contrast, the large Péclet number (Pe ≈ 5 × 10^3^) indicates that advection dominates over molecular diffusion along the channel length. This explains why baseline co-flow remains largely unmixed without the pulsed perturbation. For Device B, the oil-phase Reynolds number is very small (Re ≈ 0.05), confirming a strongly laminar microfluidic regime. The capillary number (Ca ≈ 0.012) and viscosity ratio (λ ≈ 0.013) fall within the range typically associated with stable dripping-type droplet formation in microfluidic systems.

### 3.2. Mixing (Device A)

**Figure 6:**
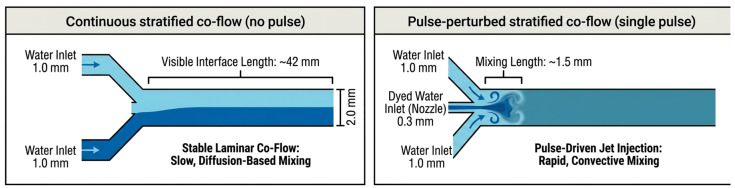
Device A mixing summary: no-pulse continuous stratified concurrent flow versus single-pulse perturbed concurrent flow.

Using the flow-state definitions in Figure 1 and Figure 6 compares Device A under (A) no-pulse steady co-flow (baseline) and (B) pulse-perturbed co-flow (single pulse). Under the reference condition, a representative visual comparison showed that the apparent mixing length decreased from ~42 mm to ~1.5 mm. This visual measure is used only as an intuitive indicator; quantitative mixing performance was evaluated using entropy-based image analysis.

**Figure 7:**
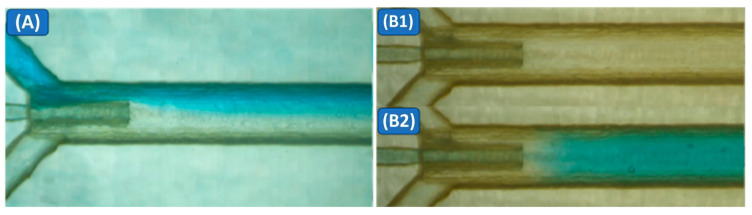
Microscopy images of mixing in Device A: (**A**) no-pulse stratified co-flow; (**B1**,**B2**) single-pulse injection producing rapid cross-sectional homogenization.

At a reference total flow rate of 500 µL/min, with no pressure pulse applied to the pulse tank, the flow remained laminar and stratified, forming a steady two-stream concurrent flow (Figure 7A). The dyed and transparent water streams met at the junction, forming a smooth, nearly straight interface clearly visible along the main channel. Downstream of the intersection, this interface broadened only slowly via molecular diffusion, without any visible vortices, recirculation, or large-scale flow disturbances, and even after several centimeters, the two regions could still be visually distinguished in the recorded images. Using channel geometry as a length reference, we found that the dyed/transparent interface remained visible for approximately 42 mm downstream of the injection point, indicating that mixing was dominated by slow diffusion in the no-pulse baseline case.

**Figure 8:**
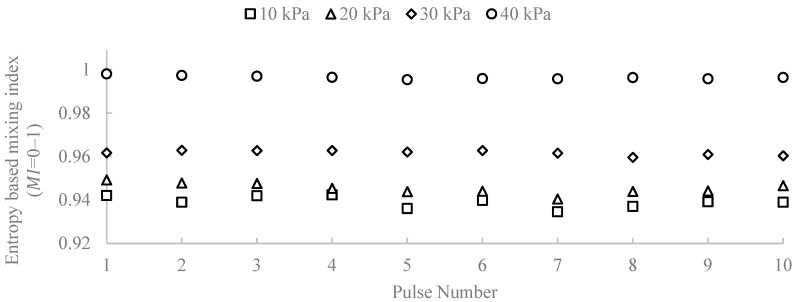
Pulse-resolved entropy-based mixing index (MI) for Device A at injection pressures of 10–40 kPa. Each point corresponds to a detected pulse event.

At the same total flow rate, applying a single 10 kPa pressure pulse of duration 0.250 s to the pulse tank introduced a transient, pulse-driven perturbation to the stratified concurrent flow behavior (Figure 7(B1,B2)). As soon as the pulse was triggered, a dyed-water jet was injected through the 0.3 mm nozzle into the main channel and penetrated across the stratified concurrent flow interface between the two aqueous streams. This central jet generated short-lived swirls immediately downstream of the junction, and the dyed fluid spread rapidly across the full channel width rather than remaining confined to one side. In the images taken after the pulse, the downstream flow appeared visually uniform across the channel width within approximately 1.5 mm of the injection point. A quantitative evaluation using the entropy-based mixing index is shown in Figure 8. Compared to the no-pulse baseline mixing length of about 42 mm, this corresponds to a reduction by a factor of roughly 28 and an approximate decrease of 96% in the distance required to reach a visibly mixed state.

**Figure 9:**
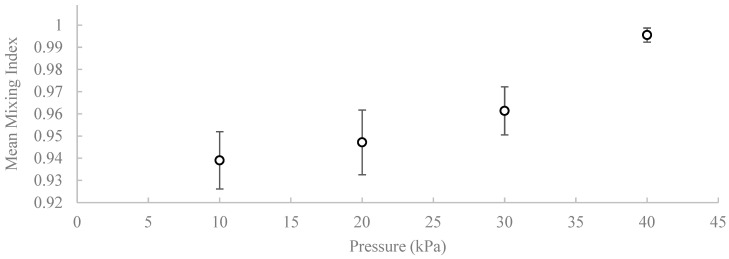
Mean entropy-based mixing index (MI) as a function of injection pressure for Device A. Values represent the mean ± standard deviation obtained from independently printed devices (*n* = 3).

The improved mixing observed after pulse actuation is attributed to transient deformation of the stratified interface and enhanced transverse transport caused by the injected pulse. Under the present conditions, increasing injection pressure increased the image-based mixing index, indicating stronger disruption of the initially stratified co-flow. A broader mechanistic and parametric analysis is left for future work. Jet injection creates a rapid disturbance in the flow, which perturbs the stratified concurrent flow and promotes interaction between fluid layers, thereby reducing the mixing length required for effective homogenization compared to traditional methods without momentum [32,33]. This disruption leads to chaotic advection, in which the fluid undergoes stretching and folding of interfaces. This physical mechanism increases the interfacial area and the stretching rate, allowing more effective mixing as fluid elements are continuously folded and stretched, thereby enhancing their interactions [34,35,36]. The chaotic advection process is crucial because it introduces complexity into the flow dynamics, which is essential for achieving efficient mixing. Moreover, enhanced convective transport occurs as the disturbances from the pressure pulse facilitate the rapid movement of fluid parcels. This improved transport mechanism accelerates mixing within the main channel, ensuring that the injected fluid is quickly integrated into the ambient flow [37,38,39,40]. In summary, the combination of jet injection, the stretching and folding of fluid interfaces, and enhanced convective transport collectively contributes to a dramatic reduction in mixing length, transforming the efficiency of fluid mixing in laminar flows [33,41,42,43]. In this study, we use a single reference operating condition for the visual mixing-length demonstration in Device A (total flow rate of 500 µL/min, 10 kPa injection pressure, and a 0.250 s pulse), selected as a representative case to illustrate the effect of pulse-driven injection on apparent mixing length. In addition, we quantify pressure-dependent mixing performance using the entropy-based mixing index (MI) across 10–40 kPa (Figure 8 and Figure 9). A broader parametric exploration of flow rate and pulse timing is left for future work. To provide an objective and repeatable evaluation of mixing performance beyond visual inspection, an entropy-based mixing index (MI) was computed from recorded RGB video frames in MATLAB R2025a. The region of interest (ROI) was defined as the channel interior covering the full observed channel length downstream of the nozzle. A nozzle location $x_{0}$ was identified in the ROI, and the analysis was applied to the downstream region ($x\geq x_{0}$). A dye-concentration proxy was extracted from each frame using a normalized blue metric (blue-channel fraction). This metric was mapped to a normalized concentration-like field c(x,y) ∈ [0,1] using two reference images: an unmixed reference (minimal dye near x_0) and a fully mixed reference (uniform dye appearance). For each downstream position x, the channel height was partitioned into $N_{w}$ bins and the bin-averaged concentration values were normalized to form a discrete probability distribution $p_{j}(x)$ ($j=1,\ldots ,N_{w}$, $\sum_{j}p_{j}(x)=1$). The normalized Shannon entropy was computed as:

(1) $$H_{norm}\left(x\right)=\frac{-\sum_{j=1}^{N_{w}}p_{j}\left(x\right)\ln\left(p_{j}\left(x\right)\right)}{\ln\left(N_{w}\right)}$$

where $p_{j}(x)$ represents the normalized concentration distribution across the channel cross-section. This entropy-based formulation has been widely used to quantify mixing uniformity in microfluidic systems [44]. With this definition, $H_{norm}=1\;$ corresponds to a uniform cross-sectional distribution across the channel height. For each frame, a downstream-averaged image-based mixing value was obtained by averaging $H_{norm}(x)$ along the downstream length ($x\geq x_{0}$). Injection pulses were identified by peak detection of a nozzle-adjacent dye signal, and the mean MI during the interior portion of each pulse was used to avoid transient edges. For each pressure condition, n=10 consecutive pulses were analyzed. Figure 8 shows the entropy-based mixing index for each detected pulse (pulse index 1–10) at nozzle injection pressures of 10, 20, 30, and 40 kPa. Within each pressure condition, the pulse-to-pulse variability is small, indicating good repeatability of the injection and mixing process. Increasing injection pressure produces a systematic increase in MI, with the highest pressure yielding near-unity mixing for all pulses.

For each frame, the entropy-based mixing index was defined as the downstream average of the normalized entropy:

(2) $$MI_{\mathrm{frame}}=\langle H_{norm}\left(x\right)\rangle_{x\geq x_{0}}$$

For each injection event, the pulse-wise mixing index was computed by averaging $MI_{\mathrm{frame}}\;$ over the interior portion of the pulse (excluding leading/trailing edges), yielding $MI_{\mathrm{pulse}}$. For each pressure condition, n=10 consecutive pulses were analyzed, and the reported values are the mean ± standard deviation over pulses.

To evaluate reproducibility across independently fabricated devices, the mean mixing index was computed for each injection pressure using data obtained from three separately printed devices. For each device, the mixing index was first averaged across the detected pulses, and the overall mean and standard deviation were then calculated across devices (*n* = 3). The resulting values are plotted in Figure 9.

As shown in Figure 9, the mean mixing index increases monotonically with injection pressure. The measured values are MI = 0.939 ± 0.0139 at 10 kPa, 0.947 ± 0.0146 at 20 kPa, 0.961 ± 0.0108 at 30 kPa, and 0.996 ± 0.0032 at 40 kPa. The relatively small standard deviations indicate good reproducibility of the pulse-driven mixing behavior across independently fabricated devices. These results confirm that increasing the injection pressure systematically enhances mixing performance under the present operating conditions.

The inclusion of measurements from independently fabricated devices indicates that the observed mixing enhancement is not specific to a single printed chip but represents repeatable behavior of the pulse-driven injection architecture.

### 3.3. Droplet Generation (Device B)

**Figure 10:**
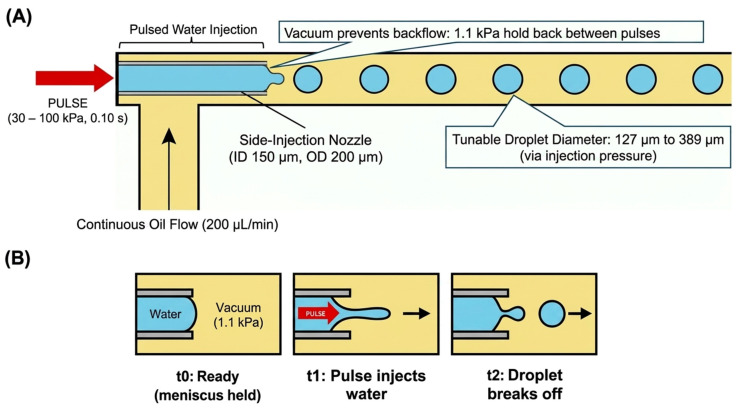
Device B droplet-on-demand principle: (**A**) nozzle placement and pulse injection with vacuum holdback, (**B**) single-pulse droplet formation sequence.

Figure 10 illustrates the operating principle of droplet-on-demand generation in Device B. Canola oil enters from the side inlet and flows past the stainless-steel nozzle tip. At the same time, short pressure pulses inject a discrete volume of water into the continuous phase. The vacuum holdback between pulses stabilizes the meniscus and prevents oil backflow into the water line. Each pulse produces a single droplet that is immediately advected downstream, and the injection pressure tunes the droplet diameter.

**Figure 11:**
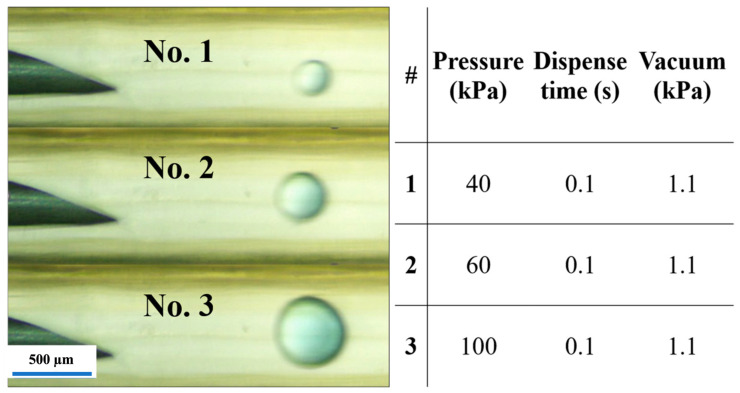
Experimental droplet generation in Device B (stainless-steel nozzle, 150 µm ID). Snapshots at 40, 60, and 100 kPa with 0.10 s pulses and 1.1 kPa vacuum holdback. Water is injected into canola oil, and droplet diameter increases with pressure (scale bar: 500 µm).

In Device B, under the typical droplet-generation conditions (canola oil at 200 µL/min in the 1.0 mm main channel and water pulses between 30 and 100 kPa with a 0.10 s duration), water-in-oil droplets formed directly at the tip of a stainless-steel nozzle protruding into the channel (Figure 11). During each pulse, a short water tongue extended from the nozzle orifice into the oil stream, then pinched off to form a single droplet, which was immediately carried downstream by the continuous oil flow. The droplets were nearly spherical and travelled as a one-dimensional train along the center of the main channel. At lower injection pressures, the droplets were smaller and more widely spaced. In contrast, at higher pressures, they became larger and more closely spaced, reflecting the increased volume of water injected per pulse. Within the pressure range used in this study, droplet formation remained in a dripping regime at the nozzle, and coalescence events were rare, while no clear transition to a continuous jet was observed.

**Figure 12:**
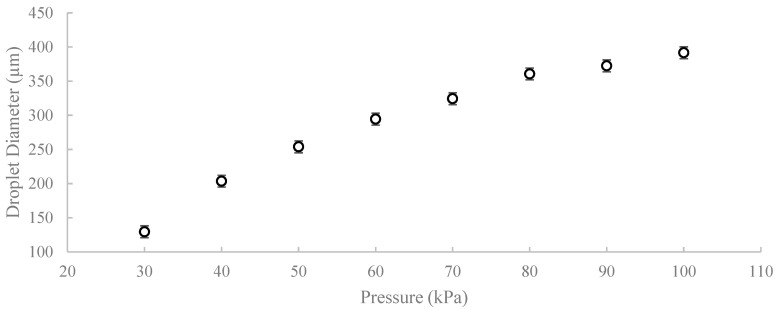
Mean droplet diameter as a function of injection pressure in Device B. Values represent mean ± standard deviation obtained from droplet measurements across independently printed devices (*n* = 30 droplets per pressure condition).

For each injection pressure between 30 and 100 kPa, droplet size was quantified by measuring the diameter of droplets observed after pinch-off, a short distance downstream of the nozzle, after stable dripping had been established. Ten droplets were measured for each independently printed device, and the measurements were combined to obtain the mean droplet diameter and standard deviation for each pressure condition (*n* = 30 droplets per pressure). The apparent droplet diameter was measured using the known 1.0 mm inner diameter of the main channel as a geometric reference to convert image distances to micrometers. Across the tested range, the mean droplet diameter increased monotonically with injection pressure, as shown in Figure 12, from approximately 129.5 µm at 30 kPa to 391.6 µm at 100 kPa. The relatively small standard deviations indicate that the pulse-driven nozzle produced consistent droplet sizes across repeated pulses and independently fabricated devices. The monotonic dependence of droplet size on injection pressure reflects the increase in injected water volume per pulse under otherwise constant operating conditions.

Under the fixed geometry and pulse duration used here, increasing injection pressure increased the injected water volume per pulse and therefore increased the resulting droplet diameter. The continuous oil phase provided shear and downstream advection for pinch-off and transport. A broader parametric analysis of pulse width, continuous-phase flow rate, and fluid properties is left for future work. As injection pressure increases, the volume injected per pulse also increases, thereby contributing to larger droplet formation. This relationship is consistent with microfluidic droplet formation, where a larger injected volume during a fixed pulse duration leads to a larger droplet before pinch-off [45,46]. Moreover, the shear and confinement created by the surrounding oil phase play a vital role in shaping the droplets. Higher pressure increases the flow rate during the pulse. Since the released volume equals the product of the flow rate and duration, and time is fixed, the total volume of water injected per pulse increases, leading to a larger droplet. This results in more controlled droplet formation, allowing the generation of larger droplets, as the confinement helps maintain the droplet structure during formation [20,47].

Additionally, droplet size dynamics are influenced by ambient conditions and nozzle geometry, further amplifying the effects of increased injection pressure. The interplay between these factors results in a complex atomization mechanism where higher pressures not only increase droplet volume but also lead to a broader distribution of droplet diameters, influencing the resulting droplet diameter distribution [45]. Thus, the combination of increased injected volume per pulse and the effects of shear and confinement at higher pressures is fundamental to understanding droplet behavior in this context.

Regarding operating limits, we found that a minimum injection pressure of approximately 30 kPa was required for reliable droplet formation. Below this value, the water emerging from the nozzle only deformed the interface slightly and then retracted, so no distinct droplets were produced. Within the explored range up to 100 kPa, droplet generation remained in a stable dripping regime, and we did not observe a transition to a continuous jet or intense breakup instabilities at the nozzle. Based on the estimated capillary number (Ca ≈ 0.012, Table 4) and the low Reynolds number of the continuous oil phase (Re ≈ 0.05), the flow remained within the laminar dripping regime typically reported for microfluidic droplet generators. A small vacuum holdback of 1.1 kPa was also necessary to maintain a stable interface between pulses. Without this feature, the pressure in the main channel can cause canola oil to slowly backflow into the pulse tank. Setting the vacuum holdback to 1.1 kPa provided a practical balance that kept the meniscus pinned at the nozzle, prevented reverse flow, and still allowed reproducible droplet ejection during each pressure pulse.

Quantitatively, under the fixed continuous-phase condition used here (canola oil flow rate 200 µL/min, main channel ID 1.0 mm, nozzle ID 150 µm, pulse width 0.10 s, vacuum holdback 1.1 kPa), droplet generation was reliable over an injection-pressure range of approximately 30–100 kPa. Within this range, droplet size was tunable. It increased monotonically with pressure, from ~129.5 µm at 30 kPa to ~391.6 µm at 100 kPa (Figure 12), with relatively small run-to-run dispersion at each condition. In this operating mode, droplet size is controlled primarily by the injected volume per pulse, which is determined by the applied pressure and pulse width. At the same time, the continuous oil flow advects the formed droplet downstream and contributes to the shear for pinch-off. Although only pressure was varied in the present study, the same platform is expected to allow further size tuning by adjusting pulse width and/or the continuous-phase flow rate.

## 4. Discussion

Integrating a pulse-driven nozzle into a straight, additively manufactured microchannel enables rapid mixing of an otherwise stratified, concurrent, laminar flow and pressure-tunable droplet generation. Compared with widely used passive chaotic-advection mixers (e.g., serpentine, staggered-herringbone, or split-and-recombine designs) and active mixers (e.g., acoustic/ultrasonic or electrokinetic mixing), the present approach emphasizes simplicity and on-demand operation rather than continuous geometric or field-driven mixing [1,6,8,10,44]. Passive chaotic mixers typically require long channels and/or complex three-dimensional features to repeatedly stretch and fold the interface, increasing footprint and pressure drop. In contrast, our device preserves an essentially straight main channel and introduces mixing only when needed via a short transverse pulse. Acoustic mixing can achieve rapid homogenization, but it typically requires external transducers, electrical driving, and careful alignment/coupling; in contrast, the present pulse-driven approach uses only air-pressure control and an integrated pulse tank, thereby reducing system complexity and being well-suited to additively manufactured devices. In the present Device A reference condition (total flow rate 500 µL/min), a single pulse reduced the visual mixing length from ~42 mm to ~1.5 mm (~96% reduction, ~28× shorter), demonstrating that substantial mixing enhancement can be obtained without complex channel designs or external fields. This result highlights the potential of pulse-triggered mixing in a straight channel. However, the visual mixing-length comparison is intended only as a representative demonstration under the present reference condition. At the same time, the present study is limited to a relatively narrow operating window and a single straight-channel architecture; therefore, broader comparison across flow rates, pulse widths, and fluid systems remains a subject for future work. The practical contribution here is the straight-channel, additively manufactured integration of a pulse tank and transverse nozzle, enabling both on-demand mixing and droplet-on-demand operation under a shared pneumatic actuation scheme. In this sense, the present platform is positioned not as a replacement for optimized conventional droplet generators, but as a structurally simple alternative to representative passive, active, and 3D-printed approaches already reported in the literature [5,11,14,16,20,21]. This combination is useful for practical lab-on-a-chip workflows that require intermittent mixing steps and/or discrete microreactors, while maintaining low fabrication complexity, straightforward scalability, and simple pneumatic control. In addition, the droplet-on-demand mode in Device B offers a practical simplification compared with conventional T-junctions and flow-focusing generators. In T-junction and flow-focusing designs, droplet size and stability depend strongly on maintaining a precise, continuous balance between the two liquid flow rates (dispersed and continuous phases) [15,16]. This typically requires two liquid pumping lines and can be sensitive to small fluctuations, start-up transients, and wetting effects, which may lead to changes in breakup behavior or droplet size. By contrast, the present device uses a single continuous oil flow at a fixed rate. It generates droplets by injecting a controlled volume of water per pulse through a transverse nozzle. The vacuum holdback further stabilizes the meniscus between pulses and suppresses reverse oil flow into the water line, improving repeatability and easing operation. As a result, droplet production is simpler to initiate, and droplet size is adjusted primarily by the pulse pressure (and pulse duration), rather than by simultaneously re-balancing two liquid flow rates. The experimentally observed droplet size range in the present work (~129.5–391.6 µm) falls within the range commonly reported for millimeter- to submillimeter-scale microfluidic droplet generators. At the same time, the straight-channel configuration reduces geometric complexity. However, previously reported 3D-printed co-flow and cross-flow systems have demonstrated smaller droplets and/or lower size variation under their respective optimized conditions, so the present work should be viewed primarily as a simpler on-demand implementation rather than a performance-maximized droplet generator [11,14]. Moreover, the present device does not yet demonstrate the very small droplet sizes or ultralow polydispersity achievable in highly optimized flow-focusing systems. Thus, the main advantage of the present platform is not absolute miniaturization or ultralow polydispersity, but structural simplicity, on-demand operation, and ease of pneumatic control in an additively manufactured format. Compared with previously reported 3D-printed co-flow, crossflow, and T-junction droplet generators, the present platform emphasizes straight-channel integration and triggerable pneumatic operation rather than the smallest reported droplet size or lowest size dispersion [11,14,16]. Likewise, compared with broader active droplet-generation approaches, the present system avoids additional field-generating components and retains a simple additively manufactured format [5]. This design also allows rapid prototyping and customization [48]. These functional observations are consistent with the estimated dimensionless numbers summarized in Table 4, which place both devices within a laminar microfluidic regime and support the interpretation of diffusion-limited baseline mixing and stable dripping-type droplet formation. Moreover, integrating a metal nozzle provides a well-defined injection orifice for repeatable pulsed droplet formation [49]. This capability is further supported by pressure-based control, which allows reproducible droplet formation under the present operating conditions [21].

## 5. Conclusions

This study demonstrates a pulse-driven, additively manufactured straight-channel microfluidic platform that achieves two key functions using only air-pressure actuation: rapid mixing enhancement in laminar co-flow (Device A) and on-demand droplet generation with pressure-tunable size (Device B). In Device A, transverse pulsed injection disrupts the stratified interface. It produces a substantial reduction in apparent mixing length relative to the no-pulse baseline. In contrast, an entropy-based mixing index computed from RGB video frames shows a monotonic improvement in whole-channel mixing and high pulse-to-pulse repeatability as injection pressure increases. In Device B, pulsed injection through a fine nozzle into a continuous oil stream yields stable dripping and repeatable water-in-oil droplets with controllable diameters across the tested pressure range.

Looking ahead, replacing the current manual/simple triggering with a programmable pressure driver would enable arbitrary pulse sequences and improve reproducibility; coupling this with image-based measurement could further allow closed-loop tuning of mixing performance or droplet size in real time. The straight-channel architecture is also well suited to multiplexed designs, such as parallel channels or multiple independently addressed nozzles on a single chip, to increase throughput and enable rapid parameter screening.

Finally, the tunable droplet-generation mode provides a practical route for applications that benefit from size-controlled microreactors, including materials synthesis and particle production. This platform is well-suited to application studies that benefit specifically from on-demand actuation in a straight channel. For example, pulse-triggered mixing can be used for time-critical assays where reagents must be combined at a well-defined moment (e.g., rapid quenching or short-time kinetic measurements), while keeping the baseline flow simple and stable between pulses. Likewise, the droplet-on-demand mode can serve as a practical microreactor format for producing discrete, size-controlled reaction volumes for materials synthesis (e.g., nanoparticles or microparticles) or for screening reaction conditions by sweeping pulse pressure (and/or pulse width) without the need to rebalance two liquid flow rates continuously. Because the device requires only one continuous-phase pumping line and pneumatic pulses for the dispersed phase (with vacuum holdback to suppress backflow), it can reduce operational complexity compared with conventional flow-focusing setups, making application experiments faster to set up and more reproducible in practice.

## Figures and Tables

**Table 1:** Comparison of the present platform against representative droplet-generation approaches.

Approach /Example	Fabrication /Integration	Inputs Required	Primary Control Method	Reported Droplet Size Range /Monodispersity	Notes
Conventional flow-focusing (framework for tuning) [15]	Rapid prototyping + framework for design/operation	2 liquid inlets (dispersed + continuous)	Flow-rate ratio, geometry, Ca	30–400 µm (reported achievable range)	Widely used baseline; continuous operation; requires balancing two flow rates
3D-printed co-flow droplet generator [11]	Micro-3D printing (projection micro-stereolithography)	2 liquid inlets	Flow rates, phase choice	CV < 3% (W/O and O/W in same device)	Demonstrates high monodispersity in printed co-flow
3D-printed “plug-and-play” crossflow [14]	3D-printed module + commercial tubing/fittings	2 liquid inlets	Flow rates	Droplets down to ~50 µm	Hybrid “printed + fittings” approach; scaling laws reported
3D-printed T-junction droplet generator [16]	3D-printed chip (T-junction)	2 liquid inlets	Flow rates, Ca	Monodispersity/polydispersity analyzed (quantitative statistics reported)	Direct 3DP version of classic geometry; validates feasibility
Geometry-set fixed-volume (“block-and-break”) [20]	T-junction + bypass channel	2 liquid inlets	Mostly geometry; less sensitive to flow variation	Fixed-volume concept (geometry-defined)	Useful comparator: droplet volume set by design rather than precise flow balance
Pressure/active droplet systems (overview) [5]	Various	Varies (often added field/actuator)	Electric/acoustic/thermal/etc.	Varies	Use as “active droplet generation” landscape reference
Pressure-stabilized droplet-on-demand driving (Y-junction) [21]	Microfluidic chip + stabilized pressure driving system	Pressure-controlled reagent bottles	Pressure waveform stability	Droplet-size distribution used to verify stability	Relevant prior art emphasizing pressure stability for droplet control
This work: straight channel + transverse pulsed injection	Additively manufactured platform + integrated pulse tank; (Device B uses inserted 150 µm ID capillary)	Device A: 2 water inlets + pulse line; Device B: 1 oil flow + pneumatic pulses for water	Pulse pressure, pulse width; (Device B) vacuum holdback	Device B: ~129.5 to 391.6 µm (30–100 kPa, 0.10 s, oil 200 µL/min; present study)	Emphasizes straight geometry + shared actuation for mixing + droplets

**Table 2:** CAD and measured diameters of accessible channel openings for printed devices (*n* = 3).

Feature	CAD Diameter (mm)	Measured Diameter (mm)	Difference (mm)
Device A main channel opening	2.00	1.82, 1.84, 1.85	−0.15 to −0.18
Device A side inlet	1.00	0.80, 0.83, 0.85	−0.15 to −0.20
Device B main channel opening	1.00	0.81, 0.82, 0.84	−0.16 to −0.19

**Table 3:** Physical properties of the working fluids used in the experiments (≈22 °C).

Property	Purified Water	Canola Oil	Basis
Density ρ (g cm^−3^)	0.9980 ± 0.0012	0.9140 ± 0.0040	Measured across 3 independent batches
Dynamic viscosity μ (mPa·s)	0.96	71.5	Estimated from the temperature correlation for water. Reference value for canola oil at ~22 °C [27]
Water–oil interfacial tension γ (mN m^−1^)	-	24.5	Literature range midpoint [24]

**Table 4:** Representative dimensionless numbers for the operating conditions used in the experiments (≈22 °C).

Device	Parameter	Value	Basis
Device A (mixing)	Channel diameter D	2.0 mm	Device geometry
	Flow rate Q	500 µL/min	Experimental condition
	Mean velocity U	2.65 × 10^−3^ m s^−1^	Calculated from Q/A
	Reynolds number Re	≈5.5	Using ρ = 0.9980 g cm^−3^ and μ = 0.96 mPa·s
	Péclet number Pe	≈5.3 × 10^3^	Using Dm ≈ 1 × 10^−9^ m^2^ s^−1^
Device B (droplets)	Channel diameter D	1.0 mm	Device geometry
	Oil flow rate Q	200 µL/min	Experimental condition
	Continuous velocity Uc	4.24 × 10^−3^ m s^−1^	Calculated from Q/A
	Reynolds number Re	≈0.054	Using ρ = 0.9140 g cm^−3^ and μ = 71.5 mPa·s
	Capillary number Ca	≈0.012	Using μ = 71.5 mPa·s and γ = 24.5 mN m^−1^
	Viscosity ratio λ	≈0.013	Using μwater = 0.96 mPa·s and μoil = 71.5 mPa·s

## Data Availability

The original contributions presented in this study are included in the article. Further inquiries can be directed to the corresponding author.
